# Versatile Peptide-Based Nanosystems for Photodynamic Therapy

**DOI:** 10.3390/pharmaceutics16020218

**Published:** 2024-02-02

**Authors:** Qiuyan Li, Ruiqi Ming, Lili Huang, Ruoyu Zhang

**Affiliations:** Institute of Engineering Medicine, School of Medical Technology, Beijing Institute of Technology, Beijing 100081, China

**Keywords:** peptide, photodynamic therapy, targeting, stimuli-responsive, self-assembly

## Abstract

Photodynamic therapy (PDT) has become an important therapeutic strategy because it is highly controllable, effective, and does not cause drug resistance. Moreover, precise delivery of photosensitizers to tumor lesions can greatly reduce the amount of drug administered and optimize therapeutic outcomes. As alternatives to protein antibodies, peptides have been applied as useful targeting ligands for targeted biomedical imaging, drug delivery and PDT. In addition, other functionalities of peptides such as stimuli responsiveness, self-assembly, and therapeutic activity can be integrated with photosensitizers to yield versatile peptide-based nanosystems for PDT. In this article, we start with a brief introduction to PDT and peptide-based nanosystems, followed by more detailed descriptions about the structure, property, and architecture of peptides as background information. Finally, the most recent advances in peptide-based nanosystems for PDT are emphasized and summarized according to the functionalities of peptide in the system to reveal the design and development principle in different therapeutic circumstances. We hope this review could provide useful insights and valuable reference for the development of peptide-based nanosystems for PDT.

## 1. Introduction

**Figure 1:**
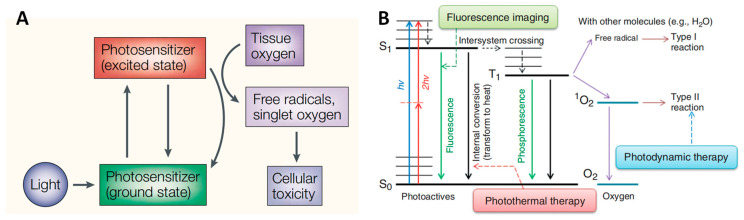
(**A**) Components and mechanism of photodynamic therapy. (**B**) Jablonski diagram illustrating the processes of fluorescence and phosphorescence decay and photothermal and photodynamic effects [12]. (*hν*, one-photon absorption; 2*hν*, two-photon absorption; S_0_, ground state; S_1_, singlet state; T_1_, triplet state.) Reproduced with permission from Ref. [11]. Copyright © 2024, Springer Nature Limited, Cham, Germany. Reproduced with permission from Ref. [12]. Copyright 2012, Wiley Periodicals, Inc, Hoboken, NJ, USA.

Photodynamic therapy (PDT) refers to the ablation of cancerous and precancerous cells by toxic reactive oxygen species (ROS) produced by photosensitizers (PSs) under light radiation at a specific wavelength, as shown in Figure 1A. PSs, oxygen, and light radiation are three indispensable components of PDT. ROS have very short lifespans (from several to hundreds of nanoseconds) and very short diffusion distances (<20 nm). Combined with the excellent spatial precision of laser radiation, PDT allows for accurate ablation without causing side effects on normal tissues. PDT has attracted intensive research interest in cancer treatment, skin disorders, and other medical conditions, as ROS has detrimental effects on all types of cells, which means it does not cause drug resistance. In addition, patients who undergo PDT often experience relatively shorter recovery times compared with those who receive other, more invasive treatment, such as resection. Therefore, PDT has been applied widely as an independent or complementary therapy for the clinical ablation of skin cancer, head and neck cancer, gastrointestinal cancer, lung cancer, brain cancer, etc. [1,2,3,4,5]. In addition, a Phase I clinical trial of PDT in patients with oral carcinoma in situ/dysplasia [6], a Phase I/II clinical trials of PDT in patients with advanced pancreatic cancer [7], and a Phase III clinical trial of PDT in basal cell carcinoma [8] have been reported on. Obviously, the optimistic outlook of PDT in clinical applications has in turn strongly encouraged the development of photosensitizers and the investigation of therapeutic methodologies to fully exploit its potential in cancer treatment.

The intrinsic photodynamic activity of PSs critically determines the efficiency of ROS generation. The porphyrin family are the most commonly used photosensitizers. Porfimer sodium (Photofrin^®^) is the first clinically approved PS for cancer treatment. Subsequently, quite a few PSs with longer excitation wavelengths and deeper penetration depths have been developed and approved by the FDA for clinical use, such as Temoporfin, Hemoporfin, Verteporfin, etc. [5]. Among these, 5-aminoketovaleric acid (5-ALA) has become a dominant PDT reagent because it can be metabolized into protoporphyrin IX (PPIX) for further ROS generation [9]. As shown in Figure 1B, a photosensitizer is excited by specific wavelengths of light and transits to the singlet excited state (S_1_). After intersystem crossing (ISC), it returns from the triplet state (T_1_) to the ground state to produce ROS. Specifically, PSs undergo type I (electron transfer) reactions to produce toxic radicals such as peroxides, superoxide anions, and hydroxyl radicals (OH•) or type II (energy transfer) reactions to yield singlet oxygen (^1^O_2_). Among ROS, OH• is the most reactive and toxic species and can damage proteins and cellular membrane structures through lipid peroxidation. ^1^O_2_ has a short half-life of 3 μs, but it can diffuse across several hundred nanometers, causing wide damage to proteins, nucleic acids, and lipids. H_2_O_2_ is relatively less reactive than other ROS, but it has the longest half-life (1 ms) and the longest diffusive distance (1 μm) among ROS. H_2_O_2_ and O_2_^•−^ undergo the Fenton reaction to produce OH• [10]. Type I PDT is believed to be more oxygen-economical, which is particularly beneficial under the prevalent hypoxic conditions in the tumor microenvironment [11].

In addition to the photodynamic activity of the PSs, the practical outcome of PDT is also determined collectively by other factors, such as oxygen concentration at the tumor lesion, the power intensity of the excitation light, etc. First, experimental results have also shown dramatic changes in the partial pressure of oxygen (pO_2_) in tissues during PDT, indicating the importance of quantifying pO_2_ for optimizing PDT efficacy [13,14]. In this regard, hybrid materials that carry oxygen-generating components or drugs that inhibit respiratory activity can be a solution to hypoxia in cancerous tissue [15]. Second, from the aspect of excitation of PSs, proper matching between the excitation wavelength and the laser beam is of great importance [16]. The excitation light is somehow attenuated by light scattering and tissue absorption by blood, skin, and lipids in biological entities, which subsequently limits PDT efficiency. Considering that the majority of tissue absorption occurs below 650 nm and above 1350 nm, infrared/near-infrared (FR/NIR) excitation facilitates deeper penetration [17]. Third, the toxicity of PSs in the absence of light irradiation (dark toxicity) is also an important safety concern. Additionally, patients who undergo PDT are usually advised to stay indoors and avoid exposure to light irradiation before PSs are completely excreted from the body. Finally, once PSs and light sources are properly selected, targeted delivery of PSs to local lesions, such as cancerous cells and tissues, is highly important. Nanomaterials carrying PSs with delicate surface modifications that enable proper circulation duration, targeted delivery, and precise release of PSs and drugs at tumor lesions are highly favorable. Nanocarriers such as polymeric nanoparticles (NPs), liposomes and hydrogels are outstanding candidates for their good biocompatibility and ease of further modifications. With different formulations and fabrication methods, the size, surface charge, and collapse behavior of nanocarriers can be fine-tuned to meet practical demands. Antibodies and ligands can be modified on the surface of nanocarriers to realize targeted delivery for PDT through interactions between antibodies and antigens, ligands, and receptors.

Peptides are chains of amino acids linked by peptide bonds with different sequences and sometimes with side-chain modifications. Peptides and proteins are both important components of biological systems. Proteins, particularly antibodies, have been widely applied in the development of targeted drugs, contrast agents, and enzyme-linked immunosorbent assay (ELISA) kits [18]. Peptides are now emerging as alternatives to protein antibodies because they are more stable, less expensive, and easier to synthesize and customize [19]. Peptides offer equivalent bioactivity but enjoy higher activity per mass. Moreover, stimuli in the tumorous environment, such as lower pH and overexpressed endogenous enzymes, can prompt peptides to change their electrostatic properties or hydrolyze somewhere in their sequence, offering the possibility of controlled release with reduced side effects [20,21,22]. With the aid of phage display technology, a great variety of peptides have been screened to show high affinity and specificity to target proteins, cells, and organelles [23,24]. Finally, by manipulating amino acid building blocks, peptides can self-assemble into stable nanoparticles/micelles which are readily to be delivered or form different self-assemblies in the tumor lesion [25]. Two or more features of peptides can be harnessed to provide synergistic or cascading functionality for targeted antitumor delivery and therapy.

**Figure 2:**
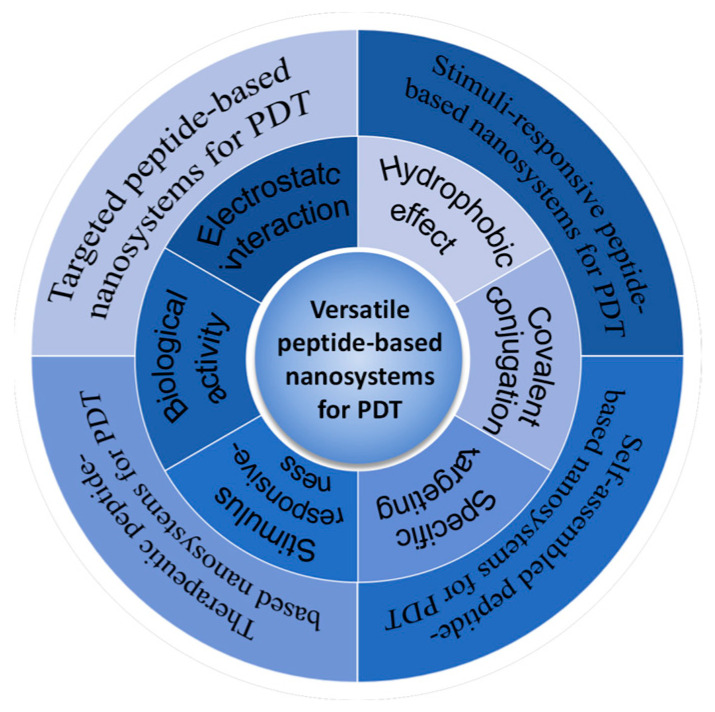
Versatile peptide-based nanosystems for PDT: from property and functionality to applications.

In this review, we started with a brief introduction to PDT and peptide-based nanosystems, followed by more detailed background information about the structure and properties of the peptides in the context of biological applications. Finally, examples of peptide-based nanosystems for photodynamic therapy have been reviewed, which were published most recently over the past five years. The design and development principles of peptides for PDT are exemplified and categorized according to their functionalities as follows: (1) targeted peptide-based nanosystems; (2) stimuli-responsive peptide-based nanosystems; (3) self-assembled peptide-based nanosystems; and (4) therapeutic peptide-based nanosystems, as illustrated in Figure 2. Examples are described in detail from the aspects of structure–property and design–application relationships to provide clues about the design and development of existing systems and inspiration for further improvement and applications.

## 2. Properties and Architecture of Peptides

### 2.1. Chemical Structures of Peptide

A peptide bond is formed between the α-carboxylic acid moiety of one amino acid and the α-amino moiety of another amino acid by losing a molecule of water through a condensation reaction. Peptides with different sequences show significant variations in physical and chemical properties. According to their side chain structure, amino acids can be categorized as positively or negatively charged (at pH 7.4), polar uncharged, or hydrophobic, as well as special cases such as cysteine (Cys), selenocysteine (Sec), glycine (Gly), and proline (Pro). All of the natural amino acids are found in two forms, the L and D enantiomers, except for the nonchiral glycine. As amino acids are the building blocks of peptides and proteins, the D and L enantiomers result in a different orientation of the substituents attached to the α-carbon and ultimately lead to different physicochemical properties and biological activity. Generally, L-amino acids are the predominant form of amino acids in living organisms. Meanwhile, peptides containing L-amino acids are subjected to natural enzymatic decomposition and thus suffer from limited stability in living organisms. In this regard, D-peptides have been developed to prevent peptides from proteolytic degradation [26].

Peptides can be categorized into linear or cyclic peptides according to their overall chemical structure. Both of them can be synthesized by solid-phase peptide synthesis (SPPS), which generally starts with the attachment of amino acids to a resin from the C-terminal residue and then proceeds to the N-terminal end [27]. Some peptides are naturally cyclic, while others are cyclized from linear precursors. One of the first natural cyclic peptides is phalloidin, found in death cap mushrooms [28]. Fluorescent phalloidin has become a commercial reagent for investigating the cytoskeleton by staining F-actin filaments [29]. In some cases, linear peptides are cyclized to improve stability. A typical example is the commonly used Arg-Gly-Asp (RGD) peptide for anticancer therapy, but it is susceptible to chemical degradation at the site of aspartic acid residue [30]. In comparison, cyclization of the RGD peptide significantly improves its solution stability by increasing the rigidity of the RGD backbone [31]. It is also noteworthy that cyclic peptides do not always offer better binding affinity than their linear precursors, which require case-by-case investigations. More recently, amino acids in peptide sequences were fluorinated to improve transmembrane ability for targeted delivery [32].

### 2.2. Function and Bioactivity of Peptide

Peptides have a diverse range of bioactivity and functionality. Peptides are essential components in physiological processes as peptide hormones, biological modulators and receptors, etc. For instance, insulin is a 51-amino acid peptide hormone that regulates the conversion of food to energy and controls sugar levels. As it consists of two polypeptide chains and has almost all the structural features of a protein, it is regarded as both a peptide hormone and a small protein. Semaglutide, a 31-amino acid lipopeptide with a linear sequence, is a peptide drug for the treatment of type 2 diabetes and weight management. It has a similar structure to the hormone glucagon-like peptide-1 (GLP-1). Another notable example is the β-amyloid peptide (Aβ), which is believed to play vital roles in the pathogenesis of Alzheimer’s disease due to the extracellular aggregation of Aβ plaques. There are also peptides that bind with metal ions and play regulatory roles in biological systems, e.g., zinc-finger peptides and peptides containing histidines or cysteines.

The bioactivity of peptides varies widely depending on their sequence, and some can be utilized independently or synergistically for cancer treatment, with properties including cell adhesion, cell penetration, and therapeutic functions [33]. In addition to the RGD peptide binding with the α_v_β_3_ integrins overexpressed in many cancer cells, PHSRN is a peptide sequence with high affinity for α_5_β_1_ integrins. The TAT (YGRKKRRQRRR) peptide is a cell-penetrating peptide derived from the transactivator of HIV transcription, which has been widely utilized as a transport vehicle for targeted delivery to both the cytoplasm and nucleus. Antimicrobial peptides (AMPs) are small peptides that have inhibitory effects on bacteria, fungi, parasites, and viruses. Typically, AMPs are cationic/amphiphilic but contain hydrophobic residues. Applications of AMPs in cancer therapy have recently been highlighted and termed anticancer peptides (ACPs), due to their anticancer activity [34]. For example, some AMPs can trigger cytotoxicity in cancer cells by binding to negatively charged phosphatidylserine moieties on the cell membrane of cancer cells. Furthermore, some AMPs, such as LTX-315, can induce release of tumor antigens and potent damage-associated molecular patterns, which is promising for immunotherapy [35]. Additionally, dolastatin 10 is an anticancer pentapeptide with antimitotic activity and an inhibitory effect on tubulin polymerization that can be conjugated with an antibody to yield anticancer drugs [36].

### 2.3. Architecture of Peptide Nanosystems

Peptides contain enriched chemical structures in their side chains, which facilitate interactions with other components or independent interactions within the peptide nanosystem. Common driving forces for these interactions include electrostatic attraction, hydrogen bonding, hydrophobic interactions, π–π stacking, etc. Peptides are frequently modified through covalent conjugation onto the surface of a broad range of nanocarriers, such as silicon NPs, lipid NPs, and Ag/Au NPs, to improve the targeting ability or stability of nanosystems [37,38,39]. Biolabeling strategies such as amine labeling, thiol labeling, azide labeling, and tetrazine labeling are common strategies for covalent conjugations because they are facile and clean and produce stable products in high yields [40].

By taking advantage of the water-solubility of peptides or further conjugating them with hydrophilic PEG chains, photosensitizers can be dispersed into aqueous media as nanoparticles or micelles. Yan’s group have considerable expertise in the field of molecular self-assembly and peptide-based nanomaterials for biomedical applications [41]. Chlorin e6 (Ce6) is a hydrophobic photosensitizer that suffers from limited solubility and instability in aqueous solutions. The cooperative self-assembly of negatively charged Ce6 and cationic diphenylalanine (CDP) has been reported to occur through hydrophobic and electrostatic interactions and π–π stacking [42], and their particle size can be tuned by altering the loading ratio of Ce6 to CDP. The assembled NPs remain stable both in aqueous solutions and in culture media, which meets the requirements for biological applications. Experimental results showed enhanced antitumor efficacy of Ce6, indicating the successful delivery of Ce6 to tumor sites via this self-assembly strategy. Additionally, peptides are employed as useful vesicles for delivery of PSs and vaccine because they are biocompatible, multifunctional, and easy to chemically modify [42,43]. For instance, Lynn et al. reported a peptide-TLR-7/8a conjugate vaccine platform for enhanced anticancer T-cell immunity [43]. A diverse array of peptide neoantigens are linked to TLR-7/8a (adjuvants), and their physical form-dependent T-cell immunogenicity was evaluated. The self-assembling behavior of the nanoparticles was fine-tuned by modifying the charge property of the peptide-TLR-7/8a conjugates to optimize the uptake and activation of antigen-presenting cells for antitumor immunity.

**Figure 3:**
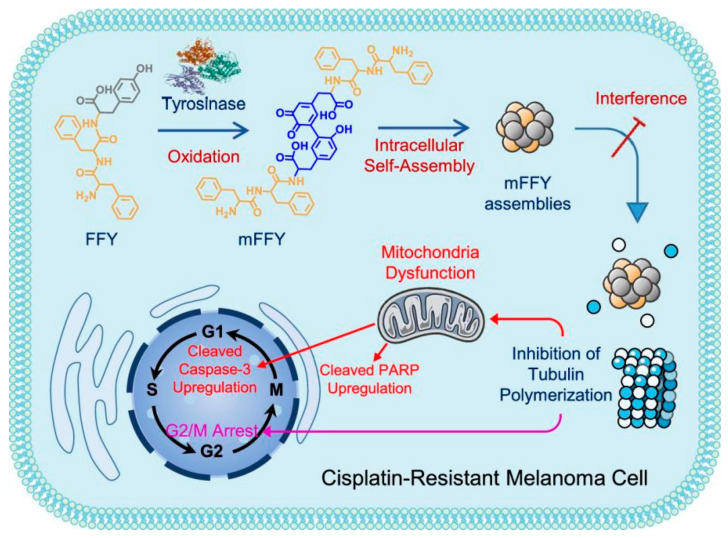
Schematic illustration of tyrosinase-induced tripeptide assemblies and their intrinsic apoptotic effects on cisplatin-resistant melanoma cells. Reproduced with permission from Ref. [49]. Copyright 2022, American Chemical Society, New York, NY, USA.

Responsive peptides are commonly shielded inside the nanocarriers until they reach the tumor lesions. Enzymes, ROS, metal ions, and pH are potential stimuli in the microenvironment to trigger the collapse of nanosystems. On-site chemical reactions or endogenous enzymatic hydrolysis in cancer cells can cleave peptide bonds or convert the charge property of peptides, resulting in precise drug release on demand [44]. On the other hand, peptides conjugated with aggregation-induced emission (AIE) fluorogens are usually designed to form aggregates upon enzymatic degradation of peptide substrates to emit turn-on fluorescence for image-guided diagnosis and therapy [45]. Intriguingly, special peptide sequences such as Phe-Phe drives peptide or peptide–PS conjugates to self-assemble into various nanostructures for cancer therapy [46]. Compared with peptide molecules, self-assembled nanostructures have advantages such as superior stability and improved enhanced permeability and retention (EPR) effects that are desirable for circulation and accumulation in tumor lesions [47,48]. In a more recent report, as shown in Figure 3, the Phe-Phe-Tyr tripeptide was delivered, intracellularly oxidized and self-assembled into melanin-like assemblies with diquinone structures. These assemblies have been proven to inhibit the self-polymerization of tubulin and induce severe G2/M arrest, inducing intrinsic apoptosis to inhibit the tumor growth of cisplatin-resistant melanoma [49]. Another study has shown that the self-assembly behavior of peptide can be modulated to change the way they interact with cells and the way they are taken up by cells. Specifically, a light-responsive molecule conjugated with hydrogen-bonding peptide moiety and targeting moiety was developed and named MQIO-P. Its molecular conformational changes and interactions with living cells were studied [50]. It was found that peptide nanofibers induced cellular apoptosis through reshaping the morphology of lysosomes and resulted in subsequent leakage of lysosomal contents into the cytoplasm. Both of these two examples emphasize that modulating peptide self-assembly can be a way to fight against cisplatin resistance.

## 3. Multifunctional Peptide-Based Nanosystems for PDT

Peptides have been widely employed in the field of photodynamic therapy for their unique features and diverse functionality. In this section, reports on versatile peptide-based nanosystems from the last five years have been collected and summarized. Examples have been categorized according to the functionalities of the peptides, including their cellular-, organelle-, and receptor-targeting abilities; stimuli-responsive activities; self-assembly properties; and therapeutic activities, such as cytolytic, anticancer, and immunotherapeutic properties.

### 3.1. Targeted Peptide-Based Nanosystems for PDT

**Figure 4:**
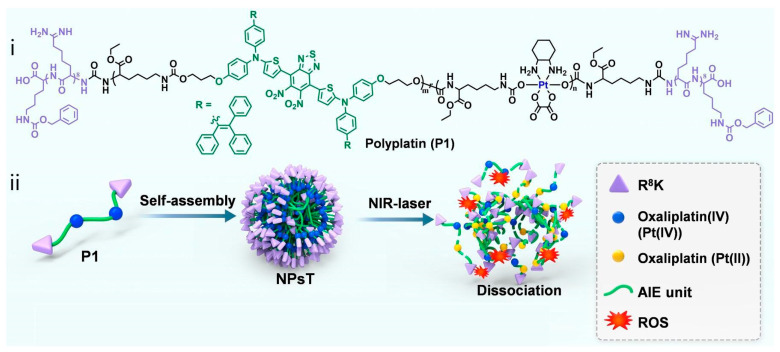
Demonstration of the nucleus-targeting Pt^IV^ NPs. (**i**) Chemical structure of the polymeric chain containing the PtIV complex, the AIE unit, and terminal cancerous tissue/nucleus-targeting peptide R8K (P1). (**ii**) Within an aqueous environment, the polymer can self-assemble into nanoparticles (NPsT). Reproduced with permission from Ref. [52]. Copyright 2022, Wiley-VCH GmbH, Hoboken, NJ, USA.

The electrostatic interactions between peptide nanosystems and the biological environment strongly affect their targeting behaviors and biodistribution. Cell-penetrating peptides (CPPs) composed of highly positively charged amino acids, such as lysine or arginine, are commonly employed to facilitate the transportation of drugs across the membranes of the cytoplasm and organelles. Wu et al. utilized the cell-penetrating peptide (Cha-Arg)_3_, which consists of three cyclohexylalanines (Cha) and three arginines (Arg), to deliver the natural photosensitizer riboflavin (RF, vitamin B2) with improved cellular uptake [51]. Chemotherapy has long suffered from significant toxicity to normal cells and tissues, which emphasizes the importance of targeted delivery and on-site activation of prodrugs such as Pt^IV^. Recently, Wei and Xiao et al. reported that NIR-activatable nanoparticles self-assembled from a polymer chain integrated with the Pt^IV^ prodrug oxaliplatin, a PS with AIE characteristics, and the cell-penetrating peptide R_8_K, as shown in Figure 4 [52]. The cationic R_8_K peptide allows the polymeric chain to self-assemble into spherical nanoparticles, which accumulate effectively in the nucleus of cancer cells. Under 808 nm light irradiation, the metal center of the Pt^IV^ was selectively reduced to Pt^II^, which resulted in degradation of the nanoparticles, while the AIE photosensitizer was activated to produce ROS and induce immunogenic cell death (ICD). Other attempts have also been made to improve the EPR effect by conjugating photosensitizers with amphiphilic polypeptides [53]. The targeting ability of the peptide R_8_K greatly minimizes the severe side effects of the Pt^II^ drug. Lin et al. conjugated PPIX with the pH-dependent peptide WHHHFFHFFHFFHFF (P12) to yield the new compound PPIX-P12. The histidine and phenylalanine residues allow PPIX-P12 to bind firmly to tumor cell membranes under acidic conditions, resulting in improved cancer cell ablation efficiency [54].

ROS have very short lifespans and very high toxicity, which demands targeted delivery and precise activation at local lesions to optimize therapeutic outcomes. The plasma membrane is essential for maintaining normal physiological activities. Since biological lipid bilayers are negatively charged, cationic/amphiphilic (hydrophilic and hydrophobic) peptides are frequently employed to improve membrane-targeting ability. For example, PpIX has been conjugated with peptide sequences containing different amounts of arginine (Arg) or glutamic acid (Glu) to modulate the plasma membrane-targeting ability of photosensitizer–peptide conjugates. The results showed that even one arginine could help to anchor PpIX to the plasma. By conjugating four Arg residues to PpIX, the resultant PpIX-Arg4 could self-assemble into spherical nanoparticles, which are preferentially taken up at tumor sites through the EPR effect [55]. PpIX-amphipathic peptide-PEG chain automatically self-assembled into spherical micelles in aqueous solution, which facilitated tumor accumulation after tail vein injection. The experimental results also demonstrated that dual-targeting peptides greatly improved PDT efficacy by initiating cell apoptosis and necrosis through a decrease in the mitochondrial membrane potential and disruption of the plasma membrane.

**Figure 5:**
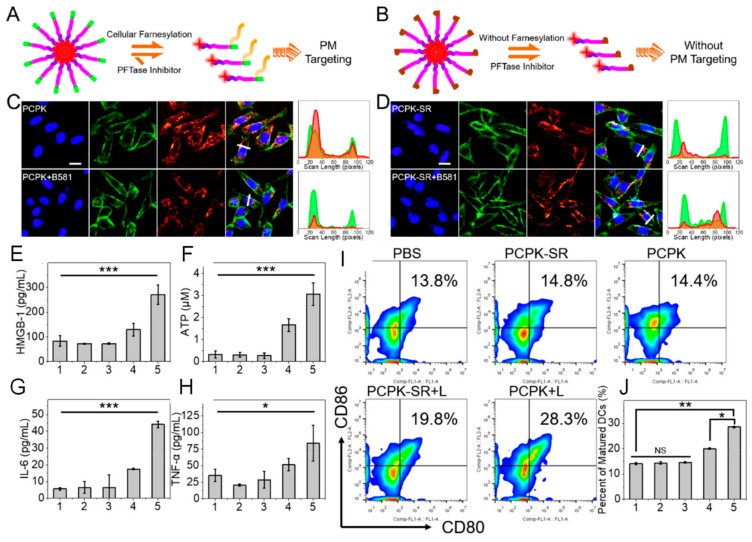
Schematic illustration of (**A**) PCPK with PM-targeting ability endowed by cellular farnesylation processes and (**B**) PCPK-SR without PM-targeting ability by inhibited cellular farnesylation processes and the in vitro validation of (**C**) PCPK and (**D**) PCPK-SR by fluorescence imaging costained with the commercialized membrane tracker CellMask green plasma membrane. Levels of (**E**) HMGB-1 and (**F**) ATP in the cell supernatant and in vivo cytokine detection of (**G**) IL-6 and (**H**) TNF-α in sera from mice after different treatments. (1: PBS, 2: PCPK-SR, 3: PCPK, 4: PCPK-SR + L, and 5: PCPK + L; L indicates 660 nm light irradiation (LED light, 30 mW cm^−2^). (**I**) In vivo detection of DC maturation (CD80^+^ CD86^+^) in response to different treatments by FACS and (**J**) quantitative analysis of mature DCs. Significance was calculated via one-way ANOVA with a Tukey posthoc test. * *p* < 0.05, ** *p* < 0.01, *** *p* < 0.001. NS represented no significant difference. Adapted with permission from Ref. [57]. Copyright 2019, American Chemical Society.

Zhang’s group have considerable expertise in the field of peptide-based biomedical applications. For example, an amphiphilic chimeric peptide, rFxrFxrFxr, with dual-targeting affinity for mitochondria and the plasma membrane, where r represents D-arginine and Fx represents L-cyclohexylalanine, has been reported on [56]. A protein known as farnesyltransferase (PFTase)-driven plasma membrane-targeted chimeric peptide (PCPK) was reported to guide photosensitizers to specifically damage the plasma membrane (PM) [57]. PCPK has obvious membrane-targeting ability compared with that of PCPK-SR, which lacks membrane targeting, as shown in Figure 5A–D. Rupture of PM was shown to result in rapid release of damage-associated molecular patterns (DAMPs), triggering enhanced antitumor immune responses compared with cytoplasm-localized PDT, as shown in Figure 5E–J. In combination with anti-PD-1 immunotherapy, the PM-PDT strategy had an inhibitory effect on distant metastatic tumors. The cationic amphipathic peptides that were first designed, such as (KLAKLAK)_2,_ have good binding affinity to mitochondrial and plasma membranes [58]. Additionally, TAT is one of the most widely applied cell-penetrating peptides for targeted imaging and therapy of cancer cells [59,60,61]. The nuclear localization sequence NLS peptide (PKKKRKV) was engineered on the surface of exosomes to facilitate dual-targeted delivery at both the plasma membrane and nucleus [62].

**Figure 6:**
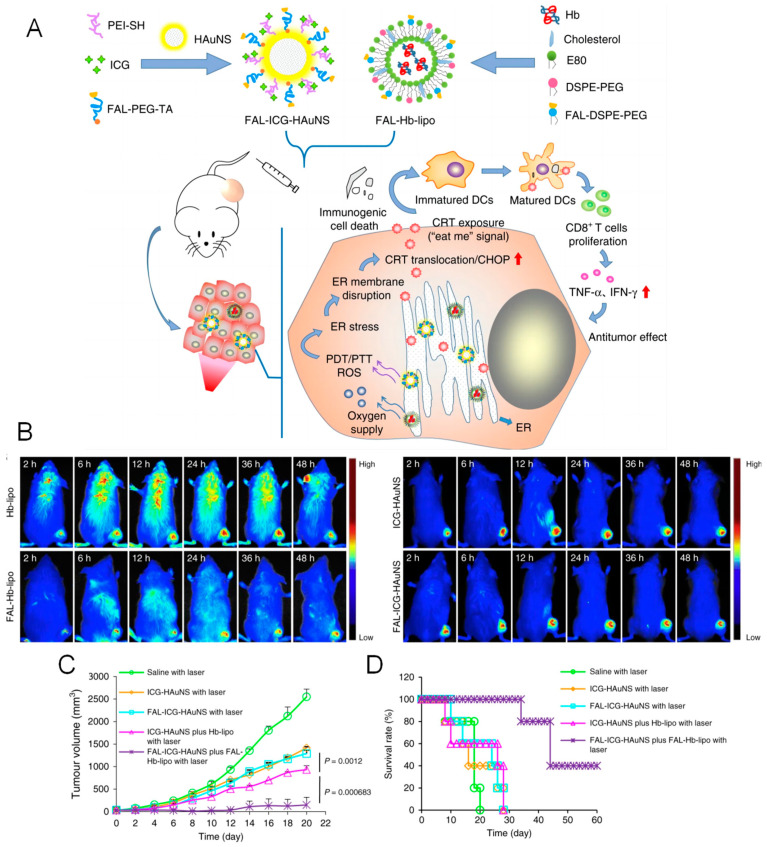
The antitumor mechanism of FAL-ICG-HAuNS plus FAL-Hb-loaded liposomes. (**A**) Schematic illustration of enhanced immunogenic cancer cell death and anticancer effects induced by ER-targeting photothermal/photodynamic therapy. (DC: dendritic cell; CHOP: C/EBP-homologous protein-10, an ER apoptotic protein). (**B**) Biodistribution of Hb-lipo, FAL-Hb-lipo, ICG-HAuNS, and FAL-ICG-HAuNS after intravenous injection. (**C**) Volume of tumors in groups with different treatments as indicated in the figure. (**D**) Survival rate of tumor-bearing mice receiving different treatments as indicated in the figure. Reproduced with permission from Ref. [64] under a Creative Commons Attribution 4.0 International License.

Activation of the immunogenic cancer cell death (ICD) process has emerged as an important strategy for fighting against therapy-resistant cancer, while endoplasmic reticulum (ER) stress and ROS play vital roles in regulating immunogenicity through the release of DAMPs [63]. ER-targeted PDT and photothermal therapy (PTT) were developed to activate ICD [64,65]. Specifically, the ER-targeted peptide pardaxin (FAL) and indocyanine green (ICG) are both conjugated into hollow gold nanospheres to yield nanosystem FAL-ICG-HAuNS, as shown in Figure 6. Gold nanospheres are promising photothermal therapeutic agents and ICG produces ROS, so FAL-ICG-HAuNS can be used for synergistic PDT and PTT. Under near-infrared (NIR) light irradiation, FAL-ICG-HAuNS induces strong ER stress and exposure to calreticulin (an immunogenic cell death marker, [CRT]) to stimulate antigen presentation in dendritic cells, thereby activating a series of immune responses. The level of hypoxia in the tumor environment severely limits the production of ROS during PDT. Therefore, hemoglobin (Hb) liposomes can be used to deliver oxygen to the ER to reverse hypoxia, ensuring the efficacy of PDT/PTT. According to in vivo experimental data, NP-conjugated FAL showed improved tumor-targeting efficiency and better tumor retention than those without conjugated peptide FAL, as shown in Figure 6B. The survival rate of the tumor-bearing mice and the tumor volume shown in Figure 6C,D demonstrate the enhanced antitumor effect by PTT/PDT achieved by the co-delivery of FAL-ICG-HAuNS and the FAL-Hb-loaded liposomes through ER-targeted immunogenic cancer death and relief of hypoxia in tumor lesions. In addition to ICD induced by ER stress, PDT targeting other organelles such as the cell membrane and mitochondria have been reported. A membrane-targeting chimeric peptide named CCP has been reported for PDT ablation of tumor cells [66]. Specifically, positively charged RRKK fragments of CCP have a high affinity for membranes through electrostatic interactions, while the alkyl chain of palmitic acid promotes its insertion into the cellular membrane. Next, the plasma membrane is disrupted in situ by ROS generation, releasing cellular contents such as DAMPs, which triggers an intense ICD effect. Similarly, mitochondria-targeted PDT has been reported to effectively induce the release of DAMPs in cancer cells and the final ICD, probably because of mitochondrial dysfunction [67]. In addition, some anticancer peptides such as oncolytic peptides and antimicrobial peptides have come to be regarded as ICD inducers by promoting tumor-specific cell lysis and immune stimulation [68]. A self-assembling EGFR-targeting peptide was conjugated with an AIEgen to yield TPA-FFG-LA to form nano-assemblies on the surface of EGFR-positive triple-negative breast cancer (TNBC) cells, which inhibit EGFR signaling and trigger lysosomal membrane permeabilization (LMP) after being internalized into the cells [69]. Upon light irradiation, AIEgens produced large amounts of ROS, exacerbated LMP, and triggered strong ICD.

**Figure 7:**
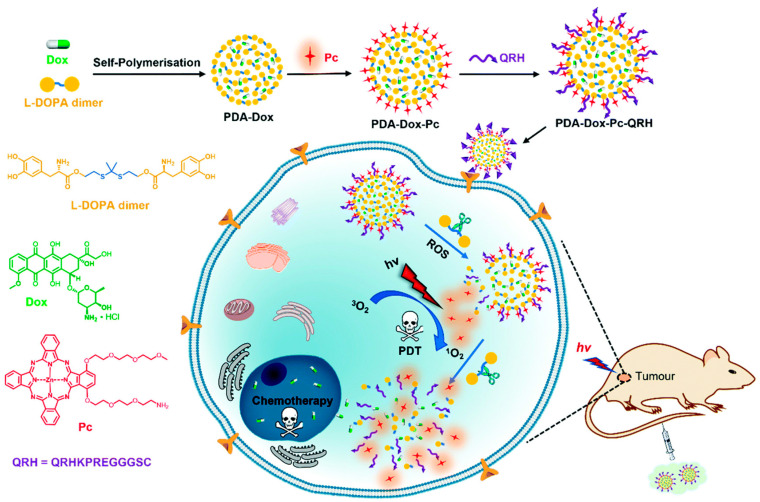
Schematic illustration of the preparation procedure of PDA-Dox-Pc-QRH NPs and the working mechanism for synergistic PDT/CDT anticancer treatment. Reproduced with permission from Ref. [71]. Copyright 2021, The Royal Society of Chemistry, London, UK.

Targeted delivery to cancer cells by ligand/receptor interactions is also a powerful strategy for anticancer therapy. Receptors that are overexpressed on cancer cells increase the accumulation of ligand-functionalized nanosystems in tumors and effectively reduce tumor growth relative to that in normal tissues. Commonly used ligand/receptor pairs include T7-peptide/transferrin, folic acid/folate, and α_v_β_3_ integrin/RGD [70]. Additionally, peptide QRHKPREGGGSC (QRH) preferentially binds to epidermal growth factor receptor (EGFR) cancer cells. As shown in Figure 7, both QRH and a zinc (II) phthalocyanine (Pc) photosensitizer are conjugated on the surface of polydopamine (PDA) NPs which encapsulate DOX and are preinserted with ROS-cleavable thioketal linkers between dopamine molecules [71]. Pc on NPs was inactive under normal physiological conditions due to the self-quenching effect of stacked Pc molecules and the quenching effect of PDA. Upon uptake of this nanosystem by EGFR-positive breast cancer cells, endogenous ROS in the tumor region trigger the disassembly of NPs by cleaving thione bonds and releasing doxorubicin (DOX) and Pc molecules. Under NIR light irradiation, the Pc molecules generate additional ROS, which further accelerate the cleavage of the NPs. In this example, both EPR effect and the EGFR-targeting peptide contributed to targeted delivery to cancer cells, and the subsequent ROS-activated photosensitizer and release of DOX in tumor lesions further improved the dimensional precision of both PDT and chemotherapy, minimizing the side effects on normal tissues.

Polyhedral oligomeric siloxane (POSS) molecules are used as drug carriers due to their unique nanostructure and superior biocompatibility. POSS has been conjugated with Ce6 and the polypeptide 18-4 (WxEAAYQrFL) to achieve targeted PDT via recognition of the breast cancer surface receptor keratin 1 (KRT1) [72]. Upconversion nanoparticles (UCNPs) can be excited by multiple low-energy photons, avoiding the toxicity induced by short-wavelength light, which is favorable for biological applications. Liposomes encapsulating UCNPs and PS methyl blue (MB) have been surface-modified with an anti-HER2 peptide for the ablation of HER-2 positive breast cancer cells [73]. The anti-HER2 liposomes showed enhanced PDT efficacy against HER2-positive cells, such as SKBR-3 cells, compared with HER2-negative MCF-7 cells, which emphasizes the importance of peptide-targeting ability. Many other ligand/receptor pairs have been reported for targeted PDT, and the results are summarized in Table 1.

### 3.2. Stimuli-Responsive Peptide-Based Nanosystems for PDT

**Figure 8:**
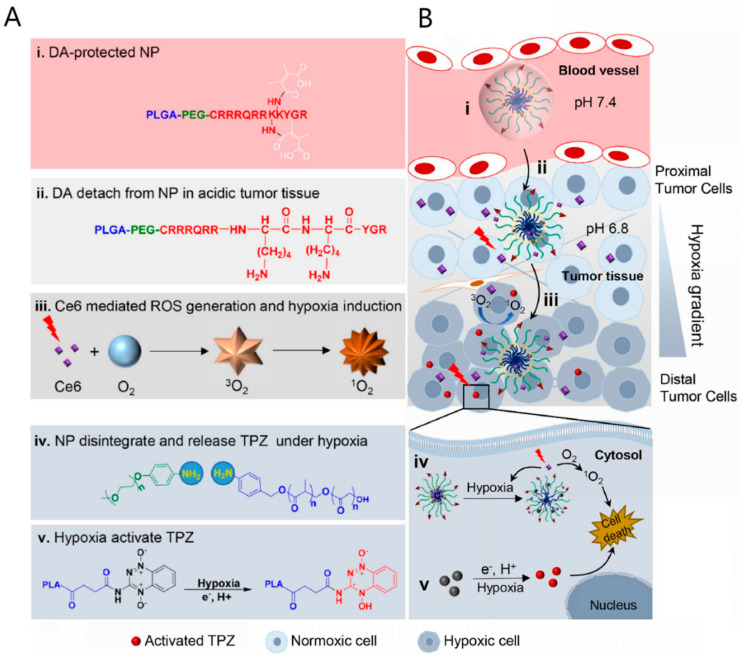
(**A**) Schematic illustration of the designed structure and functionality of TAT + AzoNPs. (**i**) DA-protected NP allows for stable circulation. (**ii**) DA is detached from NP in acidic tumor tissue. (**iii**) Under light irradiation, Ce6 produces ROS and induces hypoxia in tumor. (**iv**) NP disintegrate and release TPZ under hypoxia. (**v**) TPZ is activated under hypoxia condition.(**B**) the proposed anticancer mechanism through stepwise−activatable hypoxia−triggered PDT. (**i**) pH 7.4 in blood vessel. (**ii**) pH 6.8 in tumor tissue. (**iii**) hypoxia condition gradually become severe as oxygen is consumed to produce ROS. (**iv**) toxic ROS induces cell death. (**v**) activated TPZ induces cell death under hypoxia in cytosol. Reproduced with permission from Ref. [59]. Copyright 2020, Elsevier Ltd., Amsterdam, The Netherlands.

Stimulus-responsive peptide-based nanosystems could be designed by the following strategies to facilitate targeted delivery and controlled release of PSs: (1) integration of peptide sequences containing chemical bonds vulnerable to stimulus or enzymatic degradation and (2) utilization of peptides with charge-convertible properties upon stimulation of tumor lesions. Stimuli that can be utilized to trigger the conduction of photodynamic therapy include light, pH, enzymes, glutathione (GSH), and ROS. By encapsulating PS Ce6 and a hypoxia-activatable prodrug tirapazamine (TPZ) in a polymeric matrix and modifying the surface with a protected TAT, acidity-activatable nanoparticles (NPs) were reported, as shown in Figure 8A,B [59]. The acidic environment of the tumor triggered amide bond breakage, exposing TAT peptides at the tumor site for enhanced accumulation. Moreover, the TPZ precursor was modified with hypoxia-sensitive azobenzene bonds, which can be activated in the hypoxic tumor microenvironment. Based on a light-responsive ruthenium (II) polypyridyl compound, a prodrug for both chemotherapy and PDT with RGD as targeting peptide was reported [83]. The ruthenium ligand bond is shielded in the dark, while under light irradiation, the ruthenium-based drug is released because of the photo-substitution properties of the ruthenium polypyridyl complex exerting significant enhanced toxicity on tumor cells. The photo-substitution is also accompanied by significant ROS generation. Collectively, this prodrug represents a promising strategy for phototherapeutic treatment.

**Figure 9:**
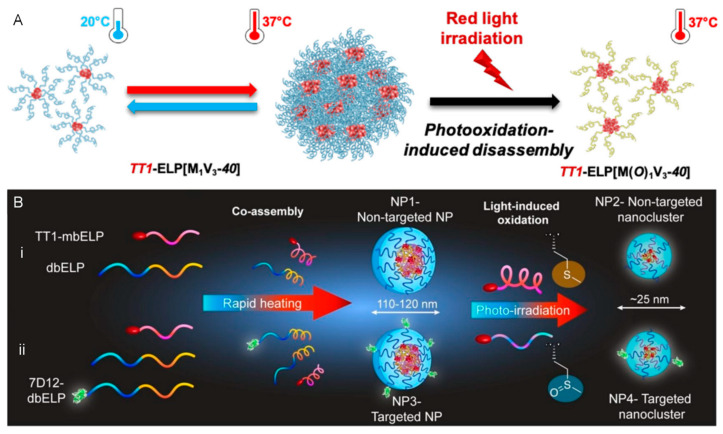
(**A**) Proposed mechanism of the assembly and disassembly behaviors of the temperature and photooxidation-responsive nanosystems containing elastin-like polypeptides (ELPs). (**B**) Schematic of the formation of co-assembled NPs without (**i**) and with (**ii**) the EGFR-targeting nanobody 7D12. Upon light irradiation, photo-oxidation can rapidly disassemble the Met-containing ELP-based NPs into smaller nanoclusters. Conjugation of 7D12 nanobodies endows the co-assembled NPs with EGFR-targeting ability. Reproduced with permission from Ref. [84]. Copyright 2021, American Chemical Society. Reproduced with permission from Ref. [85]. Copyright 2023, Wiley-VCH GmbH.

Elastin-like polypeptides (ELPs) are commonly used temperature-responsive polypeptides. These artificial protein polymers are composed of repetitive sequences of GXGVP pentapeptides. These particles exhibit hydrophobic properties above the critical aggregation temperature (cloud point T_CP_) at a given concentration. Using methionine (Met, M) and valine (Val, V), a singlet oxygen-responsive hydrophobic ELP scaffold was developed. Because the thioether group of methionine is easily oxidized to sulfoxide, hydrophobic ELP with a low T_CP_ can be transformed into a hydrophilic sulfoxide derivative with a high T_CP_. Next, the constructed ELP scaffold was conjugated with Zn(II)-phthalocyanine PS (denoted as TT1). Under light irradiation, the ROS generated by the photosensitizer TT1 oxidize ELP into sulfoxide derivatives. Increased hydrophilicity results in a change from aggregated micelles to single micelles, which makes it easier for the particles to diffuse into dense tumors and improve the effectiveness of PDT treatment, as shown in Figure 9A,B [84]. More recently, a light-responsive nanoplatform has been reported has having enhanced tissue penetration and an improved antitumor effect. Methionine residues were integrated in TT1-ELP monoblock conjugates and self-assembled with diblock ELP into an ELP-based nanoparticle, which changed rapidly from 120 nm to 25 nm under photo-irradiation and exposure to singlet oxygen produced by a photosensitizer. EGFR-targeting nanobody 7D12 have been used for targeted delivery, and together with their smaller size, the 25-nm NPs can penetrate deeper into spheroids and kill cancer cells more efficiently [85].

The acidic microenvironment at the tumor site can induce a shift from a negative to a positive charge on the peptide, which enhances cellular internalization and intracellular drug accumulation. pH- and redox-responsive nanoparticles have been reported that use pH-responsive dimethylmaleic anhydride (DMMA) to bind thiol-modified polylysine (PLL) while loading ICG for PDT/PTT [86]. After nanoparticles enter the tumor microenvironment, the DMMA layer is exfoliated, and because of the high concentration of glutathione in tumor microenvironment, NPs zeta potential is reversed to a positive value, which enhanced cellular uptake and cytotoxicity upon light irradiation. Radiotherapy is still the most commonly used therapeutic strategy in clinical applications, which encourages scientists to develop X-ray-induced PDT. Since copper-cysteamine (Cu-Cy) nanoparticles can be efficiently activated by X-rays to generate ROS, a low-pH-sensitive peptide pHLIP was conjugated to yield pHLIP-Cu-Cy NPs for tumor-targeted delivery with enhanced tissue penetration [87]. In addition, since the concentration of ROS in the tumor microenvironment is much greater than that in normal tissues, ROS-sensitive chemical groups can be integrated into nanoparticles. Commonly used ROS-sensitive groups include sulfides, selenides, tellurides, diselenides, thiokals, etc. [88]. A pH/ROS dual-responsive peptide prodrug assembled by the host–guest recognition of pillar [5] arene (P5) has been reported [89]. The prodrug consists of the following parts: (1) a charge-reversable moiety 2,3-dimethylmaleic anhydride (DMA)-conjugated P5-poly(lysine), which undergoes charge conversion from negative to positive in acidic tumor lesions due to the hydrolysis of DMA; (2) a DOX-conjugated poly(lysine) with a reactive ROS-responsive thioketal (TK) bond, which releases toxic DOX under high-level ROS; and (3) through host–guest recognition, the former two parts can form precursors that further encapsulate Ce6 to yield the supramolecular polypeptide prodrug SPP-DOX/Ce6. Both the acidic and ROS-upregulated environments in the tumor region trigger the release of DOX and Ce6, and the subsequent ROS generated by Ce6 further accelerate the collapse of the prodrug. This example represents a common design of stimuli-responsive prodrugs for controlled photodynamic/chemo-cancer therapy.

Tumor regions are featured with upregulated enzymes, including cathepsin B, caspases, and matrix metalloproteinases. By integrating enzyme-responsive peptide substrates into the nanosystem, precise drug release can be achieved at tumor sites upon enzymatic hydrolysis. Cathepsin B expression is upregulated in a variety of human cancers. A GFLG peptide that can be cleaved by cathepsin B is conjugated to the BODIPY dimer BDP-BDP-NH_2_. Under normal physiological conditions, due to the electron-donating amino group and peptide conjugation, the intramolecular charge transfer process is blocked, preventing the production of singlet oxygen. However, after the GFLG peptide was cleaved by cathepsin B, the amino group was restored, and the PDT effect of BDP-BDP-NH_2_ was activated [90]. Studies have shown that combined therapy is more effective than single therapy. Therefore, a prodrug micelle based on a triple-sensitive multifunctional polymer (including a pH-/enzyme-/singlet oxygen reaction) capable of achieving synergistic PDT/chemotherapy (CT) was designed [91]. The micelles were connected to the amphiphilic copolymer PMPC and polylysine (PLL) with the GFLG peptide. Moreover, the hydrophobic chemotherapeutic drugs GEM and dimethylmaleic anhydride were grafted onto PLL by singlet oxygen-sensitive bonds (BATA) and pH-sensitive imine bonds, respectively. Finally, the photosensitizer Ce6 was encapsulated in the micelles by self-assembly of the amphiphilic copolymer. After the micelles entered the tumor microenvironment, the surface changed from negatively charged to positively charged, which promoted cell uptake. The GFLG peptide was degraded by cathepsin B in lysosomes to release free Ce6, which then produced singlet oxygen under light to induce GEM release and activate chemotherapy. On the other hand, mitochondria damaged by singlet oxygen produced a large amount of ROS, which would also kill tumor cells.

**Figure 10:**
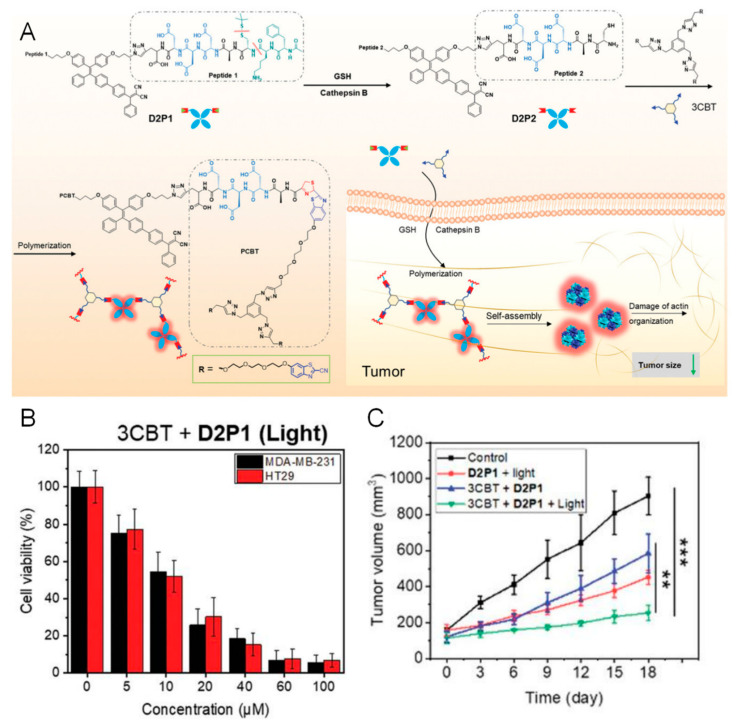
(**A**) Schematic illustration of peptide AIEgen D2P1 and its enzyme-mediated intracellular reduction and condensation with 3CBT, which result in an enhanced fluorescence signal and tumor treatment efficacy. (**B**) Cell viability of MDA-MB-231 and HT29 cells treated with 3CBT and D1P1 in the presence of light irradiation. (**C**) The volumes of tumor from groups with different treatment as indicated in the figure.(The level of significance was defined at ** *p* < 0.01, *** *p* < 0.001). Reproduced with permission from Ref. [92]. Copyright 2021, Wiley-VCH GmbH.

To achieve high accumulation of small-molecule probes at the tumor site, small-molecule probes can be assembled into macromolecules or nanostructures at the tumor site through bioorthogonal reactions (such as azide cycloaddition reactions and tetrazine–cycloene cycloaddition reactions). Qi et al. designed a molecule that can be condensed in situ to form aggregates by using the condensation reaction between cysteine and cyanobenzothiazole (CBT) [92]. The molecule consisted of D2P1 and 3CBT, as shown in Figure 10A. D2P1 includes a peptide that can be cleaved by cathepsin B and a protected side-chain cysteine that can be reduced by GSH and a covalently conjugated AIEgen. After D2P1 and 3CBT entered the tumor cells, the peptide was cleaved by cathepsin B, which triggered the GSH-induced condensation of cysteine and 3CBT, thereby self-assembling into nanoaggregates in situ, and the fluorescence signal was enhanced. Moreover, the residence time of the aggregates in the tumor was prolonged, which enhanced the therapeutic effect of photoconductive therapy. As shown in Figure 10B, the cell viability of both MDA-MB-231 cell and HT29 cell decreased significantly upon the treatment with 3CBT and D1P1 in the presence of light irradiation, while those treated with D2P1 or 3CBT alone, or treated with both but in the absence of light irradiation, showed negligible killing of cancer cells. The results indicate the effectiveness of the light-triggered PDT and the good biocompatibility in the absence of light. The in vivo experimental results shown in Figure 10C further support the success of this enzyme-mediated PDT.

Cell apoptosis is mediated through the caspase-3/7 pathway, which allows for manipulation of PDT through caspase-3-responsive peptide [93]. The DEVD peptide sequence, which can be specifically cleaved by caspase-3, is used to bind photosensitizers to gold nanoparticles [94]. The fluorescence of the photosensitizer is quenched by the gold nanoparticles under normal conditions, and the photosensitizer is released only after the peptide is cleaved, which prevents unwanted PDT and thus protects the skin from phototoxicity during treatment. The DEVD sequence can be cleaved by caspase-3 for detection of apoptosis. Additionally, it is protonated in the acidic tumor microenvironment, showing increased hydrophobicity, which yields larger nanoparticles for accelerated cell internalization and tumor retention [95]. Matrix metalloproteinase-2 (MMP-2) has also been reported to be an important biomarker for the early diagnosis of cancer, and the MMP-2-cleavable peptide EGPLGVRGK can be used as a switch for nanoprobes for precision therapy [96]. Su et al. prepared pH/enzyme dual-sensitive polymeric micelles, and in the tumor microenvironment at low pH and high MMP-2, these micelles sequentially triggered the shedding of PEG and the release of the PD-L1 antibody, thus realizing synergistic immunophotodynamic therapy [97]. In addition to pH/MMP-2, ROS-responsive moiety can be inserted to develop pH/ROS/MMP-2 triple-responsive drug nanocarriers to achieve chemotherapy/photodynamic combination therapy [98].

### 3.3. Self-Assembled Peptide-Based Nanosystems for PDT

The self-assembly of peptide nanoparticles can be divided into two categories. One is those self-assembled before delivery to endow the nanosystems with good stability in aqueous media and maintain good structural integrity during in vivo cycling for biological applications. Fmoc-L3-OMe is a simple leucine derivative with good hydrophilicity and biocompatibility, and it is also a commonly used short peptide for self-assembly. Coassembly of porphyrin PS with short peptides such as Fmoc-L3-OMe and Fmoc-L3-Arg have been reported to form NPs with good biocompatibility and photodynamic activity [99,100,101]. Amphiphilic peptides, which usually consist of a hydrophobic tail and a hydrophilic peptide head, can self-assemble into nanoparticles under specific conditions to encapsulate photosensitizers [102]. A nanoparticle self-assembled from the amphiphilic hexapeptide L_4_K_2_ linked to the fluorescein molecule FL has been developed to achieve photodynamic therapy at tumor sites [103]. The C_18_GR_7_RGDS peptide at the hydrophilic (RGDS) and hydrophobic (C_18_) ends of the R8 spacer can also be prepared as an amphiphilic engineered peptide, and the photosensitizer can be encapsulated on this basis, which can be used for combination therapy with SDT-PDT-PTT [104]. Peptides rich in cationic residues (e.g., Lys and Arg) promote the self-assembly of hydrophobic PSs into spherical nanoparticles through electrostatic interactions to enhance the accumulation of drugs at the tumor site [105,106].

Supramolecular self-assembly has become an attractive way to construct nanomaterials with a variety of structures, the driving force of which mainly relies on noncovalent interactions such as hydrogen bonding, host–guest interactions, charge transfer, and metal coordination. Spherical micelles can be formed by self-assembly between α-cyclodextrin-linked Ce6 and polyethylene glycolated peptides through host–guest complexation [107]. Dendritic peptide is a commonly used supramolecular self-assembly material. A supramolecular assembly constructed from a polyethylene glycol-modified dendritic peptide conjugate (PDPP) has been reported [108]. By adjusting the concentration of PDPP, supramolecular molecules can self-assemble to form nanomaterials of different sizes and shapes via noncovalent interactions. To improve the stability of self-assembly, pillararenes can be incorporated into peptide assemblies, and self-assembly behavior can be generated through the host–guest interaction of pillararenes and temperature control. Compared with traditional covalent peptide modifications, this supramolecular peptide does not require any purification and has the advantages of simple preparation, strong stimulation reactivity, and strong controllability [109].

**Figure 11:**
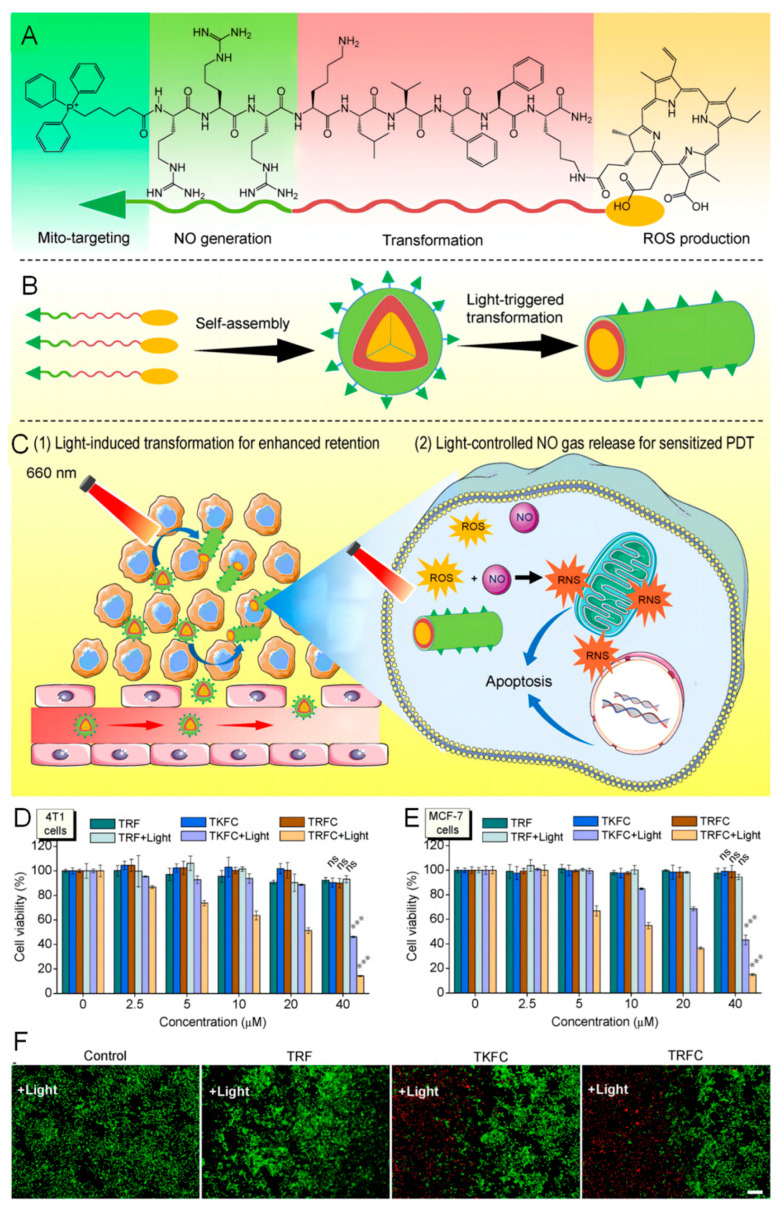
Schematic illustration of the light-triggered NO generation and structural transformation of peptide-based NPs for enhanced intratumoral retention and sensitizing PDT. (**A**) Molecular structure of the TRFC peptide monomer. (**B**) Schematic illustration of the self-assembly and in situ light-triggered nanosphere−to−nanorod structural transformation of TRFC NPs. (**C**) Schematic illustration of the structural transformation required for enhanced intratumoral retention and the mechanism of NO gas-sensitized PDT treatment. Cell viability of (**D**) 4T1 cell and (**E**) MCF-7 cell treated with TRF, TKFC, and TRFC NPs in the presence/absence of light irradiation. (**F**) LIVE/DEAD fluorescence images of 4T1 cells treated with TRF, TKFC, and TRFC NPs in the presence (**left**)/absence (**right**) of light irradiation. The level of significance was defined at *** *p* < 0.001. Reproduced with permission from Ref. [112] under the CC BY-NC-ND license.

Meanwhile, peptide self-assembly forms nanostructures at the tumor site under specific physiological environments or upon a specific stimulus. For example, some peptide molecules are prone to forming nanofibers in acidic environments, which can induce cancer cell death. By using short peptides or amphiphilic amino acids as substituents, various self-assembled photosensitizer nanosystems can be prepared. A representative self-assembling peptide sequence is phenylalanine-phenylalanine (FF), a frequently used peptide derived from Alzheimer’s disease [110]. FFs and their derivatives are also frequently used for the supramolecular construction of a wide variety of nanostructures, such as nanotubes and nanofibers [111]. A chimeric peptide nanoparticle (TRFC, TPP-RRRKLVFFK-Ce6) was reported for light-triggered nitric oxide (NO) release and structural transformation for antitumor therapy, as shown in Figure 11A–C [112]. First, photosensitizer Ce6 is hydrophobic and can form nanospheres together with covalently conjugated hydrophilic moiety TPP-RRR in an aqueous medium. When irradiated by light, ROS are generated by Ce6 and oxidize RRR domain to release NO gas, which drives further structural transformation of the KLVFF (Lys-Leu-Val-Phe-Phe) domain from nanospheres to nanorods with enhanced intratumoral retention ability. Additionally, NO gas can be further oxidized by ROS and further converted into peroxynitrite anions (ONOO^−^) with higher cytotoxicity for antitumor therapy. TRF NPs (without photosensitizer Ce6), TKFC NPs (without NO donor structure) were set as control nanoparticles. As shown in Figure 11D,E, in the absence of light irradiation, TRF, TKFC and TRFC NPs exhibited little cytotoxicity and low cytotoxicity against cancer cells. In contrast, under light irradiation, the cell viability of cancer cells declined upon treatment of TRFC NPs in a dose-dependent manner. The Live/dead fluorescence staining results also support the success of TRFC NPs in cancer cell ablation, as shown in Figure 11F. In some cases, FFVLK peptide can promote nanosytem to form nanofibers for enhanced retention inside tumor [113]. Additionally, the acidic tumor microenvironment can also induce the nanosystems self-assemble into micellesto increase the accumulation of photosensitizers at the tumor site [114]. The pentapeptide FF-Ampf-FF (AmpF) is developed by conjugation of FF with a pH-sensitive moiety 4-aminoproline (Amp), which form superhelcial and nanoparticles under neutral and mild acidic pH conditions, respectively [115]. The nanoparticles facilitated penetration and accumulation at tumor sites, while the superhelcial morphology favored the blood circulation and tumor retention. Sun et al. developed a peptideporphyrin PS by conjugation of a pH-responsive dipeptide tryptophan–glycine (WG) to hydrophobic porphyrin (P) cores, which self-assembled into nanoparticles under normal physiological conditions but transformed into nanofibers due to the driving force of enhanced intermolecular hydrogen bond formation under acidic environment [116]. The protonation of porphyrin has higher ^1^O_2_ generation rate, and the nanofibers endowed the PS with higher accumulation and long-term retention at tumor sites.

### 3.4. Therapeutic Peptide-Based Nanosystems for PDT

Peptides with therapeutic bioactivity can be utilized for antitumor therapy. Antimicrobial peptides (AMPs) play an important role in fighting against bacterial infections, and researchers have found that some of them also possess anticancer activity by inducing apoptosis, immune cell recruitment and other mechanisms [117]. Conjugation of AMP with photosensitizers to achieve synergistic treatment can enhance the antitumor effect. For example, when conjugated with AMPs such as magainin, buforin, and apidaecin, hydrophobic PSs exhibit better targeting efficiency against tumor cells and enhanced ablation rate of cancer cells [118]. However, rapid renal clearance and high systemic toxicity are common concerns and a hindrance in applying AMPs in antitumor therapy. Therefore, albumin is adopted as matrix to encapsulate AMP- pyropheophorbide-a (PPA) conjugate, to enhance the stability of AMPs in biological system and to solve the problems of hemolysis and organ dysfunction caused by AMPs [119]. The (KLAKLAK)_2_ peptide is a cationic amphipathic peptide that was first designed as an antimicrobial peptide that disrupts mitochondrial and plasma membranes and initiates cell death [57]. RB is a commonly used PS which is also an ablative chemotherapy in a stage III melanoma clinical trial. After treatment with RB-mediated PDT assisted by KLAKLAK peptide, the subcutaneous tumors in C57BL/6 mice were nearly 5-fold smaller at the end of the study than those in the animals treated with RB-based PDT only, which emphasized the important roles that targeting peptides play in this nanosystem. There are many natural active peptides that have also been used in tumor therapy. Natural cyclopeptide RA-XII, isolated from rubia yunnanensis, is reported to promote cancer cell apoptosis through the AMPK/mTOR/P70S6K pathway [120]. Mellitoxin (MLT) is a nonselective cytolytic and amphipathic cationic peptide that disrupts cell membranes by forming transmembrane pores, leading to rapid cell death. Organic–inorganic scaffolds made of serum albumin (SA)-coated thin bauxite were loaded with photosensitizers and MLT, which promoted the accumulation of photosensitizer in cancer cells and facilitated the production of ROS, which further activated dendritic cells [121].

Inhibiting the programmed death-1 (PD-1)/programmed death-ligand 1 (PD-L1) pathway is a commonly used cancer immunotherapy strategy, but it suffers from drawbacks such as low stability and difficulty in modification of relevant antibodies. In this regard, anti-PD-L1 peptides (NYSKPTDRQYHF) can be used as substitutes to conjugate with photosensitizers to yield NPs for immunodynamic therapy [122]. PD-1, a major regulator of the immune response, and its ligand PD-L1 are overexpressed in several types of human cancer cells. To improve immunotherapy efficacy, PD-L1-blocking peptide (CVRARTR) and a photosensitizer can be conjugated and applied simultaneously with a transcription 3-activating factor (STAT3) inhibitor to achieve downregulation and blockade of PD-L1. This strong PD-L1 inhibition and synergistic PDT treatment significantly inhibit the malignant proliferation of tumors [123]. Synergistic immunotherapy with immune checkpoint blockade therapy and immunogenic cell death (ICD) can effectively enhance the efficacy of anticancer treatment. A nanoparticle with surface-mimicking protein secondary structures (SPSS NPs) has been reported for ICD and immune checkpoint blockade combination therapy [124]. Specifically, a PD-L1 peptide antagonist with a self-assembling motif was introduced on the surface of the photosensitizer and self-assembled into a β-sheet protein secondary structure, a conformation that can bind highly to PD-L1 while acting as lysosome-targeted chimeras (LYTACs) to mediate the degradation of PD-L1 in lysosomes. On the other hand, the central photosensitizer generates a large amount of ROS under light exposure and induces the ICD process in tumors. To downregulate PD-L1 expression, Jun Dai et al. designed GCP/*miR-140* nanoparticles through self-assembly of a caged peptide-AIEgen probe (GCP) and *miR-140* [125]. The caged peptide was degraded in a highly reducing intracellular environment and bound to MUC1 to downregulate PD-L1 expression, whereas *miR-140* directly targeted *PD-L1* mRNA, decreasing the expression of PL-L1. Moreover, the immune response at the tumor site is enhanced by the photosensitizer PyTPA-mediated PDT.

## 4. Outlook and Perspectives

The past several decades have witnessed the great impact of nanotechnology in the field of disease diagnosis and treatment [126]. On the other hand, peptides have exhibited versatile functionalities such as tunable charge properties, self-assembly behaviors, ability to target a broad range of organisms, antimicrobial activity, and other manifold bioactivities, which can be harnessed independently or integrated within nanosystems. Depending on the tumor microenvironment, diagnostic detection and responsive therapy can be achieved with the aid of peptide sequences containing cleavage sites that are subjected to endogenous enzymes and stimuli such as thiol species and ROS. Moreover, rational integration of the peptides with whole nanosystems offers chances for image-guided therapy, controlled drug release, and cascading and/or synergistic therapy. The development of phage display technology and peptide synthesis techniques, such as solid-phase peptide synthesis technology, has greatly improved peptide libraries and promoted the popularity of peptides in the field of biomedical applications. Together with aptamers, peptides have become important alternatives to protein antibodies because they are expensive, are prone to deterioration, and require strict transportation and storage conditions. The use of peptides has greatly improved the targeting efficiency of fluorogens through covalent conjugation, leading to successful fluorescence image-guided tumor surgery. Several promising candidates are being tested in clinical trials, which highlights the great potential of these peptides in biomedical research and practical applications [127]. Photodynamic therapy has been regarded as a superior therapeutic strategy because it is highly controllable, effective, and free of drug resistance. In addition to the targeting ability of peptides, therapeutic activities such as antimicrobial activity and modulatory activity for the immune response, etc., the versatile bioactivities of peptides will definitely enable photodynamic therapy with more precise ablation, fewer side effects on normal tissues, and better therapeutic outcomes. In recent years, the use of peptide nanosystems in the field of anticancer therapy has improved, but their application in humans is still in the initial stages. However, further clinical trials and additional validations still need to be completed in the future. Encouraged by the success of peptide drugs such as semaglutide, other manifold functional peptides can be utilized for anticancer applications, in addition to the most commonly used targeting ability of peptides. The current peptide-based photodynamic therapy approach is just the tip of the iceberg, but many additional peptide functionalities are going to be discovered and will benefit and push the frontiers of biomedical applications.

## Figures and Tables

**Table 1:** Receptors overexpressed in cancer cells and their targeting peptides.

Peptide Sequence (Name)	Targeting Receptor	Types of Cancer Cells	References
QRHKPRE (QRH)	Epidermal growth factor receptor (EGFR)	Lung cancer, colon cancer, breast cancer, kidney cancer, head and neck cancer, glioma, etc.	[67,74]
YHWYGYTPQNVI (GE11)			[75]
CMYIEALDKYAC (N.A.)			[76]
WxEAAYQrFL (peptide 18-4)	Keratin 1 (KRT1)	breast cancer	[72]
LQNAPRS (N.A.)	CD133	Colorectal cancer	[77]
anti-HER2 peptide	Human epidermal growth factor receptor 2 (HER2)	HER-2 positive breast cancer	[74,78,79]
cyclo-[2-NaI-Gly-d-Tyr-Arg-Arg] (FC131)	CXCR4, a cell-surface chemokine receptor	Breast cancer, ovarian cancer, lung cancer, colorectal cancer, primary brain tumors, etc.	[80]
KSD-cha-FskYLWSSK(AE147)	Urokinase-type plasminogen activator receptor (uPAR)	Aggressive cancer cells such as breast, prostate, glioma, colorectal, endometrial, bladder, liver, and melanoma cancer	[81]
KDKPPR (N.A.)	NRP-1	Glioma, acute myeloid leukemia, pancreatic cancer, lung cancer, ovarian cancer, gastrointestinal tumors, melanoma, etc.	[66]
EHWSYGLRPG (N.A.)	Gonadotropin-releasing hormone receptor (GnRH-R)	Head and neck squamous cell carcinomas	[82]
